# Exploiting NRF2‐ARE pathway activation in papillary renal cell carcinoma

**DOI:** 10.1002/ijc.35311

**Published:** 2024-12-20

**Authors:** Silvia Angori, Harini Lakshminarayanan, Amir Banaei‐Esfahani, Katharina Mühlbauer, Hella Anna Bolck, Olli Kallioniemi, Vilja Pietiäinen, Peter Schraml, Holger Moch

**Affiliations:** ^1^ Department of Pathology and Molecular Pathology University Hospital Zurich Zurich Switzerland; ^2^ Institute for Molecular Medicine Finland‐FIMM, Helsinki Institute of Life Science‐HiLIFE University of Helsinki Helsinki Finland; ^3^ iCAN Digital Precision Cancer Medicine Flagship University of Helsinki Helsinki Finland; ^4^ Science for Life Laboratory (SciLifeLab), Department of Oncology and Pathology Karolinska Institutet Solna Sweden; ^5^ University of Zurich Zurich Switzerland

**Keywords:** drug sensitivity profiles, NQO1, NRF2‐ARE pathway, papillary renal cell carcinoma, patient‐derived cells, translational medicine

## Abstract

Papillary renal cell carcinoma (pRCC) is the second most frequent renal cancer subtype but has no indicated targeted treatments. MET inhibition may be a treatment for *MET*‐driven pRCC, but there is a large group of non‐MET‐driven pRCC without targeted therapy. Activation of NRF2‐ARE pathway has been suggested to be involved in pRCC. To study the relevance of the NRF2‐ARE pathway, we characterized 60 pRCCs by copy number analysis and Whole Exome Sequencing. Because stabilisation of NRF2 results in enhanced expression of NQO1, a reductase that prevents production of reactive oxygen species, protein expression of NQO1 was analysed by immunohistochemistry (IHC) from tissue microarrays (TMAs) and by enzymatic activity assay. Finally, patient‐derived pRCC cells (PDCs) were applied for drug profiling with 18 NRF2‐ARE pathway inhibitors. We identified *MET* mutations in 5%, and mutations in four genes of NRF2‐ARE pathway (*NFE2L2*, *KEAP1*, *CUL3* and *BACH1*) in 10% of 60 pRCC samples. IHC analysis of TMAs of 638 renal cancers showed the correlation of the expression of NQO1 with poor survival outcome (*p* < .001) and high tumour grade (*p* < .001) and stage (*p* < .001) in pRCC. NQO1 mRNA, protein levels and enzymatic activity were increased in 56% of matched pRCC tissue samples and patient‐derived cells (PDCs, *n* = 9). Drug screening revealed that Brusatol and Convallatoxin are potential novel drugs for pRCC. Inhibition of NRF2 represents a novel therapeutic approach for MET‐independent pRCC patients.

## Graphical abstract

What's new?

There are no targeted treatments available yet for papillary renal cell carcinoma (pRCC). Here, the authors investigate the NRF2‐ARE pathway as a possible source of therapeutic targets. The NRF2‐ARE pathway helps the cell detect and respond to increases in oxidative stress. The authors found mutations in four genes in the NRF2‐ARE pathway in 10% of pRCCs tested, and they also found that overexpression of the reductase NQO1 correlated with worse survival and higher tumor grade and stage. Finally, drug screening results suggested that inhibiting Nrf2 could be a potential targeted treatment for pRCC.

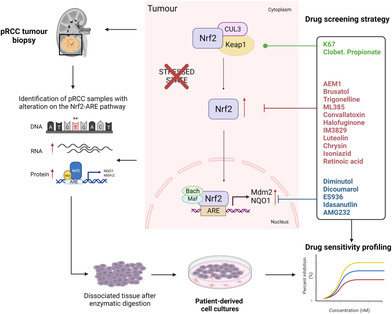

## 1 INTRODUCTION

The NRF2‐ARE (Nuclear factor erythroid 2‐like Related Factor 2—Antioxidant Response Element) pathway represents one of the most important cellular pathways for sensing and responding to increases in oxidative stress.^1^ Regulation of the NRF2 pathway is mediated by the binding of the transcription factor NRF2 to its cytosolic repressor KEAP1 (Kelch‐like ECH‐associated Protein 1). In unstimulated condition, KEAP1 targets NRF2 for proteosomal degradation, while under oxidative stress condition, NRF2 promotes the transcription of many genes involved in energy metabolism, cell‐cycle regulation and apoptosis.^2^, ^3^ In cancer cells, aberrant NRF2 activation promotes tumour initiation, progression and resistance to chemotherapeutics.^4^ Stabilization of NRF2 results in enhanced expression of NQO1 (NAD[P]H Quinone Dehydrogenase 1), an important tumour‐associated reductase that under physiological circumstances prevents the production of ROS.^5^, ^6^

Papillary Renal Cell Carcinoma (pRCC) is the second most frequent histologic subtype of renal cancer, accounting for up to 15% of all RCC.^6^ There is evidence that dysregulation of MET (hepatocyte growth factor receptor) signaling pathway plays a role for pRCC,^7^ with MET inhibitors showing efficacy only in *MET*‐driven pRCC.^8^ However, as we found only 5% of our sample harboring *MET* mutations, this highlights the medical need of effective therapies in the larger group of non‐MET‐driven pRCC.^9^, ^10^ Previous studies have shown NRF2 activation in pRCC.^11^, ^12^, ^13^ Abundant mRNA expression of the NRF2‐ARE pathway genes was associated with decreased survival in pRCC, suggesting that deregulation of the NRF2‐ARE pathway is involved in pRCC progression.^14^, ^15^ Here, we aimed to clarify the potential relevance of the NRF2‐ARE pathway in pRCC by studying its association to the pRCC patient survival using tissue microarrays (TMA), genetic aberrations by whole exome sequencing (WES), RNA‐seq and its functional activity in pRCC patient‐derived cell cultures (PDCs). In addition, we conducted drug screening of pRCC PDCs with NRF2‐ARE pathway inhibitors to explore if they represent a promising treatment option for pRCC patients.

## 2 MATERIALS AND METHODS

### 2.1 Patients and tissue samples

Two tissue microarrays (TMAs) from FFPE tissue samples of 638 RCC (clear cell RCC, papillary RCC and chromophobe RCC) and paraffin‐embedded cell blocks from patient‐derived cell cultures (PDCs) were prepared as previously described.^16^ Additional FFPE tissue samples from 60 pRCC patients were identified in the tissue biobank of the Department of Pathology and Molecular Pathology of the University Hospital Zurich (Zurich, Switzerland) while survival data were acquired from the Zurich Cancer Registry. The tumours were histologically classified according to the 2022 WHO classification.^17^ Detailed clinico‐pathological information about the tumours is shown in Table 1. Fresh tissue was obtained from papillary renal cancer patients after surgery. Portions of the fresh tissue underwent snap freezing for research purposes as described^18^ and were used for generating PDCs.

**TABLE 1:** Distribution of analysed clinico‐pathologic features and outcome of 60 pRCCs.

Characteristics		pRCC
No. of patient		60
Age of patients	Mean ± SD	(70 ± 8)
	Range	41–88
Sex of patients	Male	41
	Female	19
Tumour size (cm)	Mean ± SD	(5 ± 4)
	Range	1.2–16.5
Tumour grade	Low (1–2)	30
	High (3–4)	30
Stage	I–II	52
	III–IV	8
Outcome	Dead	18
	Alive	21
	Unknown	21

### 2.2 Generation of patient‐derived cell cultures

Patient‐derived cell cultures (PDCs) were processed from freshly isolated cancer tissue samples as recently described.^19^ Briefly, cell suspension of fresh tumour and normal tissues were transferred on a collagen I‐coated cell culture flask (Corning, NY) where mitotically inactivated mouse embryonic fibroblasts (CF6_MEF, Thermo Fisher, Inc., Waltham, MA, USA) were seeded 24–48 h prior the addition of PDCs. The co‐culture was maintained in Renal Epithelial Cell Growth Medium 2 (PromoCell GmbH, Heidelberg, Germany) with 5% FBS (Gibco) in a humidified incubator at 37°C with 5% CO_2_. Cells were expanded by passaging without the addition of new CF6 feeder cells in subsequent passages. The cell culture models were validated by copy number variation analysis and whole exome sequencing compared to the original tumour tissue. All experiments were performed with mycoplasma‐free cells.

### 2.3 Immunohistochemistry

Formalin‐fixed paraffin‐embedded (FFPE) tissue sections (2.5 μm) from TMAs, pRCC tumours and PDCs were transferred to glass slides and immunohistochemically stained with NQO1 antibody (Abcam Cat# ab34173, RRID:AB_2251526). Antigen retrieval was performed using the Ventana Benchmark XT automated system and Ventana reagents (Roche Diagnostics, Rotkreuz, Switzerland). NRF2 (Novus Cat# NBP1‐32822, RRID:AB_10003994) was detected using different internal quality control mechanisms. Human breast carcinoma tissue was used as an external positive control. NQO1 antibody gave clear staining results with the protocol described above. Because stabilization of NRF2 results in enhanced NQO1 expression, NQO1 protein level can be utilized as a marker of NRF2 pathway activation. A tumour was considered NQO1 positive if the tumour cells showed unequivocal low, moderate, or high cytoplasmic expression. PRCC tumour and paired PDCs sections were compared to the normal tissue derived from the same patient.

### 2.4 Statistical TMA analysis

Contingency table analysis and Pearson's chi‐square tests were used to analyse the associations between NQO1 expression patterns and pathological parameters. Overall survival rates were determined according to the Kaplan–Meier method and analysed for statistical differences using a log rank test. A Cox proportional hazard analysis was used to test for independent prognostic information. *p* values <.05 were considered statistically significant. The statistics were performed with IBM SPSS Statistics 26 (IBM, NY).

### 2.5 DNA and RNA isolation

DNA was obtained from 60 FFPE tumour and normal tissue samples by punching 3–5 cylinders with a diameter of 0.6 mm. RNA was extracted from sections of fresh‐frozen tumour samples from nine patients (5 sections, 30 μm from each) and from the derived matched PDCs. Tumour and normal areas were marked directly on hematoxylin and eosin‐stained slides and reviewed by a pathologist. DNA extraction from FFPE tissues was performed using the Maxwell® 16 Tissue DNA/RNA Purification Kit (Promega, Madison, Wisconsin), while from frozen material the AllPrep DNA/RNA Kit (QIAgen, Hilden, Germany) was used. Concentrations of DNA and RNA were quantified by the fluorescence‐based Qubit dsDNA/RNA HS Assay Kit (Thermo Fisher Scientific, Inc., Waltham, MA).

### 2.6 Analysis of copy number aberrations (CNAs)

Affymetrix OncoScan® CNV FFPE Assay Kit (Affymetrix, Santa Clara, CA) was used to analyse genome‐wide copy‐number alterations of DNA isolated from FFPE tumour and paired normal tissues from 60 pRCC patients (IMGM Laboratories GmbH, Martinsried, Germany). The data were analysed by the OncoScan Console (Affymetrix) and Nexus Express (Biodiscovery, Inc. CA) software using the Affymetrix TuScan algorithm. DNA from PDCs (50 ng/μl in 10 μl) and from normal and tumour tissue was processed and hybridised with the Affymetrix CytoScan HD array. Briefly, Affymetrix ChAS software was used to check the sample quality and to generate the probeset files, which were further analysed with the rCGH R package (v. 3.8, Bioconductor).^20^ The absolute CNVs were determined by the rCGH analysis workflow as previously described using a normal diploid RNA reference.^18^

### 2.7 Whole exome sequencing of pRCC tissue and PDCs

DNA was extracted from FFPE tumour and paired normal tissues from 60 pRCC patients. Agilent SureSelect XT v6 + COSMIC was used for exome capture. Sequencing was done using a HiSeq 2500 instrument (Illumina, San Diego, California, USA) at the Genomic Facility Basel (ETH, Switzerland). Previously, seven pRCC PDCs were sequenced at the Functional Genomic Center Zurich (FGCZ, Zürich, Switzerland).^19^ Somatic mutations were identified in all samples using tumour‐matched normal kidney tissue for each patient. All samples underwent exome alignment using the BWA‐Picard pipeline. We used BWA‐mem to align the paired‐end reads to the human reference genome (GRCh38) and the Picard software package to mark duplicates (http://broadinstitute.github.io/picard/). GATK and Samtools were used to perform Base Quality Score Recalibration (BQSR) and indexed the aligned reads, respectively. Afterwards, variants were called with three different tools (Mutect2, VarScan2 and Strelka2). Moreover, we used SnpEff/SnpSift to annotate the identified variants. The results of each tool were filtered according to the following criteria:

1. Mutect2: ‘PASS'ed. variant
2. Strelka2: ‘PASS'ed. variants with SomaticEVS >13.01 (95% accuracy)
3. VarScan2: only somatic variant with *p* value <.1.

Next, we considered the variants that were identified at least by two tools for all the downstream data analysis. The somatic variant list was further filtered based on Cancer Gene Census (COSMIC, RRID:SCR_002260), and the TCGA results in pRCC. STRING database was used to determine the interactor patterns of *NFE2L2*, gene encoding for NRF2. The STRING database integrates protein–protein information from numerous sources including experimental findings, computational prediction methods and published data.^21^ The sequencing coverage and quality statistics for each sample are summarized in Table S6.

### 2.8 RNA Sequencing of PDCs

For RNA quantification, 5 μl of each sample was analysed using an automated RiboGreen assay on the Hamilton LabStar Instrument, with measurements performed on SpectraMax M3 using Softmax Pro 7.1.2.

The sequencing library was prepared following the Illumina Stranded mRNA Prep, Ligation Checklist (Document #1000000124519v01), using the Illumina Stranded mRNA Prep Ligation 96 Samples kit and IDT for Illumina RNA UD Indexes Set B. The library products were quantified with Agilent TapeStation 4200 using High Sensitivity DNA 1000 Kit. Samples were then diluted to 0.5 nM, pooled, and 1% PhiX (Illumina) was added. The pool was prepared for sequencing by denaturing with 77 μl of 0.2 N NaOH, incubated for 8 min, and neutralized with 78 μl of 400 mM Tris–HCl, pH 8.0. Sequencing was set up according to the Illumina NovaSeq 6000 Sequencing System Guide (Document #1000000019358v16)^2^ using a NovaSeq 6000 S4 Kit (Paired‐end 101 bp).

### 2.9 RNA‐seq data analysis

Data was demultiplexed and analysed on the Illumina DRAGEN v4.2 platform. For mapping, the Illumina DRAGEN Multigenome Graph Reference hg38 (alt‐masked_graph+cnv + hla + rna_v3) was used, and for annotation, the GENCODE Human Release 38 (Comprehensive gene annotation) was employed. DRAGEN was run in the “enable‐rna/ enable‐rna‐quantification” mode and polyG‐/polyA‐ and adapter‐trimming was activated. DRAGEN auto‐detected the ISR library type.

Mapping metrics (e.g., total reads, uniquely mapping reads, rRNA rate) were used for quality control, resulting in the exclusion of three low‐quality samples. Raw counts and TPMs from individual samples were combined into a raw count and a TPM matrix using a custom script, and gene IDs were translated to gene names. Differential gene expression analysis was performed using DESeq2 v1.42.1.^22^ The sequencing coverage and quality statistics for each sample are summarized in Table S7.

### 2.10 RT‐qPCR

RNA extraction from nine FFPE and fresh‐frozen tissues and PDC pellets was performed as described above. RNA quality was measured with RNA Qubit RNA HS Assay Kit (Thermo Fisher). cDNA was prepared using High‐Capacity RNA‐to‐cDNA Kit (Thermo Fisher). qPCR was performed using Taqman Fast Advanced master mix (Thermo Fisher) with 20 ng/μl ng of cDNA in three technical duplicates. The thermal cycler profile was as follows: 20 s at 95°C, 40 cycles of 1 s at 95°C and 20 s at 60°C. Primer and probe set assay IDs for the TaqMan assays were Hs00975961_g1 for NFE2L2, Hs00168547_m1 for NQO1 and Hs03929097 for GAPDH (ThermoFisher). mRNA expression of NFE2L2 and NQO1 were normalised to the expression of housekeeping gene GAPDH. Either normal tissue matched to the corresponding tumour, or the average value obtained from all normal tissues were used to normalize the quantitative analysis of all samples and to measure the relative increased expression of each target. The relative expression (fold change) for each sample was calculated with the ΔΔCt‐method.

### 2.11 NQO1 activity assay

Endogenous NQO1 enzyme activity in nine tumour samples and matched paired PDC was measured using a commercial NQO1 Activity Assay Kit (Abcam Plc., Cambridge, UK). In brief, 80–100 mg of fresh‐frozen material was solubilised in 500uL 1X Extraction buffer and homogenised 2 × 2 min at 20 Hz in the Tissue Lyzer (QIAgen) with 1 steel ball (QIAgen). Cell pellets, containing at least 1 × 10^6^ cells, were solubilised in 50–70 μl 1× Extraction buffer according to the size of the pellet. Both samples were then incubated on ice for 20 min and centrifuged at 18,000 g for 20 min at 4°C. Supernatants were transferred to new Eppendorf tubes and protein concentration was measured with the BCA assay method (Thermo Fisher). Samples were diluted 1:2 to the final working concentration of 100 μg/ml with Supplemented Buffer. 50 μl of each sample was plated in duplicates in 96 well plates and the reaction buffer (with or without the inhibitor) was added according to the manufacturer's protocol. The NQO1 activity was determined by following the reduction of Menadione with cofactor NADH and the simultaneous reduction of WST1 to WST1‐formazan (yellow). The activity was measured at the absorbance of 440 nm every 20 s for 30 min. The analysis was done by subtracting OD with inhibitor from OD without inhibitor and by comparing NQO1 activity in normal kidney tissue versus activity in paired tumour and PDCs.

### 2.12 Drug screening on PDCs

pRCC PDCs were cultured for 3–4 weeks on collagen‐coated T75 flasks (Corning, NY) with Renal Epithelial Cell Growth Medium (PromoCell GmbH, Heidelberg, Germany) as described before.^19^ Drug screening was performed for five pRCC PDCs and three normal kidney cell cultures. Eighteen compounds were selected based on the molecular mechanism, potential targets, on‐going clinical trials, and studies on kidney tumour and cell lines. All the compounds used in the study were diluted in DMSO, and they are listed in Table S5. 0.2% DMSO (D4540, Sigma‐Aldrich) and 100 μM benzethonium chloride (B8879, Sigma‐Aldrich) were used in each assay plate as negative and positive controls, respectively. Each compound was dissolved in DMSO and plated in five concentrations (normally within the 1–10,000 nM concentration range) on a 384‐well plate flat‐bottom (3764, Corning, NY) using ECHO acoustic dispenser (Labcyte). Plates were stored in nitrogen gas pressurized pods (Roylan Development Ltd.). 2000 cells/well were plated in 25 μl of PromoCell medium on a pre‐drugged 384‐well plates using the BRAD automatic dispenser. The plates were incubated for 72 h at 37°C in 5% CO_2_. Cell viability assays were performed by adding 25 μl of CellTiter Glo 2.0 (Promega, 1:1 volume) reagent to each well and luminescence was recorded with PHERAstar FS (BMG Labtech) or TECAN (Infinite 200 Pro) plate readers.

### 2.13 Drug screening data analysis

The BREEZE pipeline was used to analyse the drug responses.^23^, ^24^ Quality scores were created to follow the overall quality and measure the Z score of the drug screens, and EC50/IC50 and dose–response curves for each sample were generated. To assess drug sensitivity and resistance, Drug Sensitivity Score (DSS), a multi‐parametric factor derived from the modified area under the dose–response curve was calculated.^25^ The differential DSS (dDSSs) values were calculated for each drug separately by subtracting the mean DSS value of the benign samples (*n* = 3) from the PDC sample‐specific DSS of the drug. Each drug was defined as active in a PDC if the dDSS was ≥5.

### 2.14 Validatory drug screening on PDCs

pRCC PDCs were cultured for 3–4 weeks on collagen‐coated T75 flasks (Corning, NY) with Renal Epithelial Cell Growth Medium (PromoCell GmbH, Heidelberg, Germany) as described before. Drug screening was performed on three pRCC PDCs (266_C, 193_C and 195_C) in collagen‐coated flat‐bottom 6‐well plates (Corning, NY). In order to validate the therapeutic role of NRF2 inhibitors in pRCC, Brusatol, Convallatoxin, and ML385 were selected based on the drug screening. The compounds were dissolved in DMSO (D4540, Sigma‐Aldrich). 1% DMSO (D4540, Sigma‐Aldrich) was used in each assay plate as negative control. 500,000 cells/well were plated in 2 ml of Promocell medium, and treated with 200 or 400 nM Brusatol, 100 or 300 nM Convallatoxin and 100 or 300 nM ML385 after 24 h. The plates were incubated for 24 h at 37°C in 5% CO_2_, following which the cells were harvested with trypsin. Viability was recorded using Trypan Blue (15,250,061, Thermo Fisher Scientific) and cell pellet used for further protein analysis.

### 2.15 Western blot analysis

Cell pellets were lysed in RIPA buffer (R0278‐50ML, Sigma‐Aldrich) containing protease (cOmplete Mini, 11,836,153,001, Sigma‐Aldrich) and phosphatase inhibitor (PhosSTOP, 04906845001, Roche) for 1 h at 4°C. Extracted proteins were quantified using Pierce™ BCA Protein Assay (23,227, Thermo Scientific). Equal amounts of protein were loaded in a NuPAGE 4%–12% Bis‐Tris Gel (NP0323BOX, Thermo Fisher Scientific), along with SeeBLue Plus2 protein standard (LC5925, Thermo Fisher Scientific) and separated by electrophoresis followed by transfer to a PVDF membrane. The blots were used to probe our proteins of interest—NQO1 (ab34173, Abcam), MDM2 (33‐7100, Thermo Fisher Scientific) and α‐Tubulin (T9026, Sigma‐Aldrich). HRP‐conjugated anti‐mouse (62‐6520, Thermo Fisher Scientific) and anti‐rabbit (HAF008, R&D Systems) were used as secondary antibodies, and the membrane was visualized using Pierce ECL Western Blotting Substrate (32106X4, Thermo Fisher Scientific). Images were processed and quantified using ImageLab software (Bio‐Rad Laboratories). Bargraphs of normalised relative protein expression was generated using Graphpad (Prism).

## 3 RESULTS

### 3.1 NQO1 expression is frequent in pRCC

**FIGURE 1:**
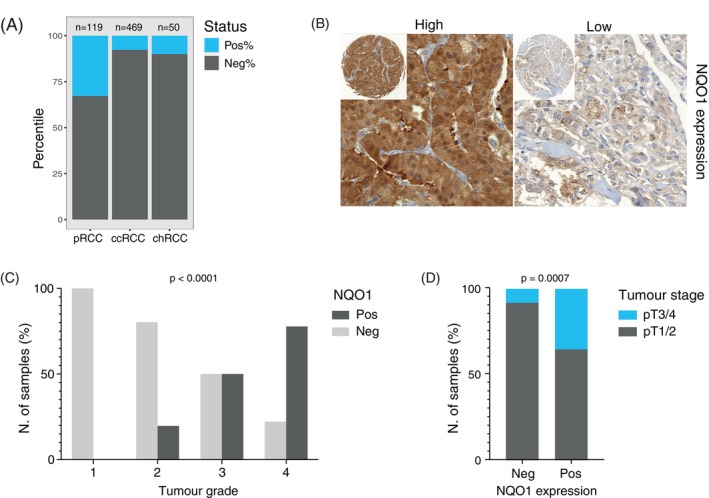
Activation of the NRF2‐ARE pathway is characteristic in pRCC. (A) NQO1 expression in TMAs of 638 RCC patient cancer tissue. (B) Immunohistochemistry of NOQ1 in pRCC:low and high cytoplasmic expression patterns (10× objective). Images of the TMA spots are included (top left corner). (C‐D) Correlation of NQO1 expression with tumour grade and stage, respectively.

NRF2 activation leads to the expression of NQO1, one of the two major quinone reductases involved in the detoxification mechanism in humans.^26^ To investigate the NRF2‐ARE pathway's activity in pRCC, we analysed NQO1 expression by IHC using TMAs that included clear cell RCC (*n* = 469), pRCC (*n* = 119) and chromophobe RCC (chRCC, *n* = 50). 32.8% (*n* = 39) of pRCC were NQO1 positive, while only 7.7% (*n* = 36) ccRCCs and 10% (*n* = 5) chRCCs showed cytoplasmic NQO1 staining (Figure 1A). Examples of low and high NQO1 expressing pRCC are shown in Figure 1B. Correlation of NQO1 expression with the clinico‐pathological features of pRCC showed that patients with NQO1‐positive tumours had a worse survival compared to those with NQO1‐negative tumours (*p* = .016; Figure S1A). Notably, all grade 1 tumours were NQO1‐negative, while the majority of NQO1‐positive tumours were high grade (50% grade 3 and 77.8% grade 4) (*p* < .0001; Figure 1C). 35.3% of advanced stage tumours (pT3/4) but only 8.1% of low stage tumours (pT1/2) were NQO1‐positive (*p* = .0007; Figure 1D). Although the fraction of NQO1 positive ccRCC was small (36 of 469 tumours), we found significant association of NQO1 expression with high ISUP grade (*p* = .0045) and worse patient survival (*p* = .003) (Table S1 and Figure S1B). No correlations were seen in chRCC (data not shown). Multivariate Cox regression analysis indicated that positive cytoplasmic NQO1 is an independent predictor in RCC (pRCC and ccRCC combined, *p* = .007; Table S2).

### 3.2 Key genes of the NRF2‐ARE pathway are mutated in pRCC

**FIGURE 2:**
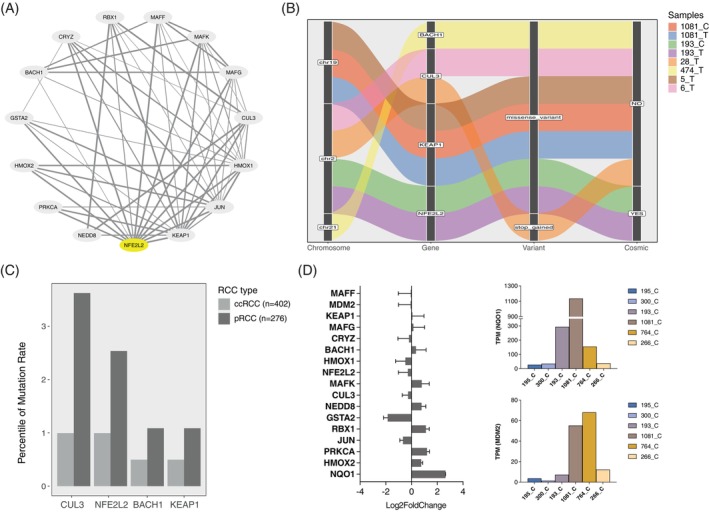
Interactions and mutations of key genes of the NRF2‐ARE pathway in pRCC. (A) STRING network view of NFE2L2 (NRF2), KEAP1 and CUL3 and their interactions. (B) Alluvial plot of four mutated genes of the NRF2‐ARE pathway in six of our pRCC samples and in the corresponding PDC (T = tumour, C = PDC). (C) Mutation frequency of the four key genes of the NRF2‐ARE pathway in pRCC and ccRCC based on the TCGA data. (D) Differential gene expression analysis of NRF2‐regulated genes and transcripts per million (TPM) of NQO1 and MDM2 in six pRCC PDCs.

In addition to the TMA samples, we performed CNV analysis in 60 pRCC. All tumours showed multiple chromosomal gains, mainly of chromosomes 7 (74.3%), 16 (77.1%) and 17 (88.6%), the most characteristic chromosomal alterations in pRCC^27^ (Figure S2). Using the STRING database, 15 interacting proteins involved in the NRF2‐ARE pathway were identified^28^ (Figure 2A). Next, we performed WES analysis to select MET‐independent pRCC samples characterised by mutations in the key genes of the NRF2‐ARE pathway identified by STRING analysis. First of all, we identified *MET* mutations in 3 of 60 samples (5%). None of these samples harbour mutation in the NRF2‐ARE pathway. In addition, six of 60 (10%) pRCC showed mutations in 4 of the 15 key genes (Figure 2B and Table S3) of the NRF2‐ARE pathway. All the NRF2‐ARE pathway mutations were independent of MET mutations. *CUL3* was mutated in two samples. Three tumour samples presented mutations in *NFE2L2* (gene encoding for NRF2) or *KEAP1*. Interestingly, the *NFE2L2* missense variant c.246A > C identified in sample 193_T, is annotated in the catalogue of somatic mutation in cancer (COSMIC database, COSV67960116) as pathogenic in different cancer types including lung adenocarcinoma, renal cell carcinoma and liver carcinoma (Tables 2 and S3).

**TABLE 2:** Characteristics of the COSMIC mutations on *NFE2L2* and *Keap1* identified in a subset of pRCC samples.

COSMIC ID	Genes	AA mutation	CDS mutation	Exon	Domain	Pathogenic
COSV67960116	NFE2L2	p.E82D	c.246A > C	2	Neh2	Yes
COSV50635496	KEAP1	p.R362W	c.1084C > T	3	Kelch	Yes

*Note*: The consequences of the mutations are described at the cDNA level in the affected exons and at the protein level with the switch in the amino acid sequence. (AA: Amino acids, E: Glutamic acid, D: Aspartic acid, R: Arginine, W: Tryptophan, CDS: Coding sequence, A: Adenine, C: Cytosine, T: Thymine, Neh: Nrf2 ECH homology 2).

In addition, we investigated the mutation rates of *KEAP1*, *NFE2L2*, *BACH1* and *CUL3* in 678 RCCs in the TCGA data. In pRCC, their mutation rates were higher compared to ccRCC. For example, CUL3 is mutated in 3.5% (10 of 276) pRCC but only in 1% (4 of 402) ccRCC (Figure 2C). The mutation frequencies in the four genes observed in our pRCC cohort (12%) and in TCGA (8%) were similar indicating that the NRF2‐ARE pathway is affected by mutations in approximately 10% of pRCC.

Finally, we conducted RNA‐seq analysis on nine normal PDCs and six tumour PDCs, focusing specifically on NRF2‐regulated genes listed in Table S4. Differential gene expression analysis showed that *NQO1* had the highest log2 fold change among all the other key NRF2‐ARE pathway genes. Moreover, we observed high transcripts per million (TPM) values for *NQO1* in three PDCs and for *MDM2* in two PDCs (Figures 2D and S5). Notably, PDCs 1081_C and 193_C, which had the highest TPM for *NQO1*, also harboured missense mutations in *KEAP1* and *NFE2L2*, suggesting a potential link between genomic alterations in the NRF2‐ARE pathway and NQO1 overexpression.

### 3.3 NQO1 is activated in pRCC patient‐derived models

**FIGURE 3:**
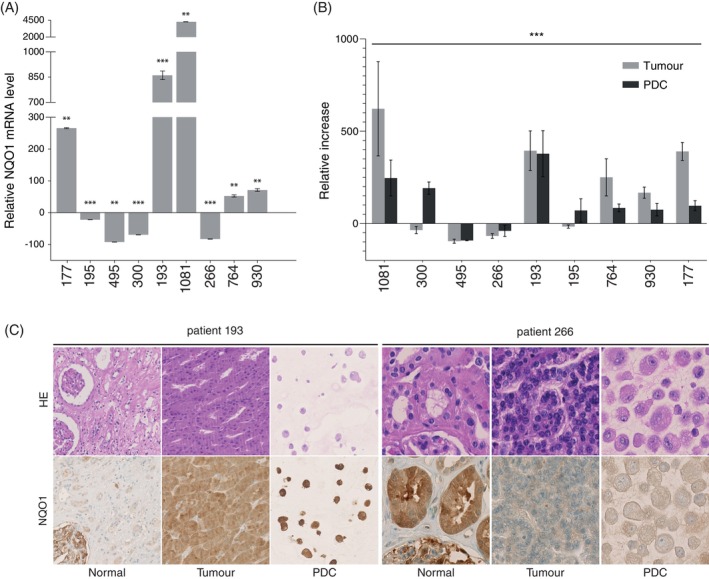
NQO1 overexpression in pRCC samples and paired PDC. (A) qPCR analysis of NQO1 in pRCCs (*n* = 9). The relative expression was normalized based on the matched normal kidney tissue (when present) or on the average values of normal tissue (**2 replicates, ***3 replicates). (B) NQO1 activity assay in matched tumour and PDC culture lysates. Results were normalized using paired normal kidney tissue when available or the average value of all the normal kidney tissues (*n* = 7). (C) Examples of NQO1 staining of pRCC PDC and the corresponding normal and primary tumour tissue samples. Sample 193 showed NQO1 overexpression in the tumour compared to the normal tissue. This over‐expression was also confirmed in the corresponding PDC model. Sample 266 did not show any significant NOQ1 over‐expression in the tumour and in the corresponding PDC. The images were taken using a 10× objective.

To further explore aberrations of the NRF2‐ARE pathway in pRCCs, we analysed the mRNA expression of *NFE2L2* and *NQO1* in nine PDC models derived from primary pRCC. While *NFE2L2* mRNA levels were similar among the samples and lower compared to the normal tissues (Figure S3A), *NQO1* mRNA was over‐expressed in five pRCC samples (55.6%) compared to the normal kidney tissue (Figure 3A). Among these five samples, whole exome sequencing data revealed that two PDC models, 193_C and 1081_C presented the same missense mutations in *NFE2L2* and *KEAP1* as found in the paired tumour tissues (Figure 2B).

Next, we analysed NQO1 activity and expression in pRCC and matched paired PDC compared to normal tissue. Five pRCC PDCs (1081, 193, 764, 930 and 177) showed increased activity of NQO1 compared to the normal kidney tissue (Figure 3B). Only two pRCC PDC (300 and 195) showed a discrepancy between the tumour and the cell culture: while the *NQO1* mRNA level (Figure 3A) and activity (Figure 3B) in the tumour were downregulated compared to the normal, the PDC showed increased NQO1 activity. Notably, five tumour tissue samples with high *NQO1* mRNA levels showed NQO1 activation, which was confirmed in the corresponding PDC.

To validate the aberrant level of NOQ1, we evaluated NQO1 protein expression in the original tumour samples. IHC analysis showed NQO1 over‐expression in the five tumour tissues and PDCs (474, 193, 764, 177 and 1081) while we did not detect any significant difference in NRF2 staining (Figures 3C, S3B and S4). Taken together, our results show that a subgroup of pRCC is characterised by NQO1 over‐expression which suggest, hyper‐activation of the NRF2‐ARE pathway.

### 3.4 NRF2‐ARE pathway inhibition as a pRCC treatment strategy

**FIGURE 4:**
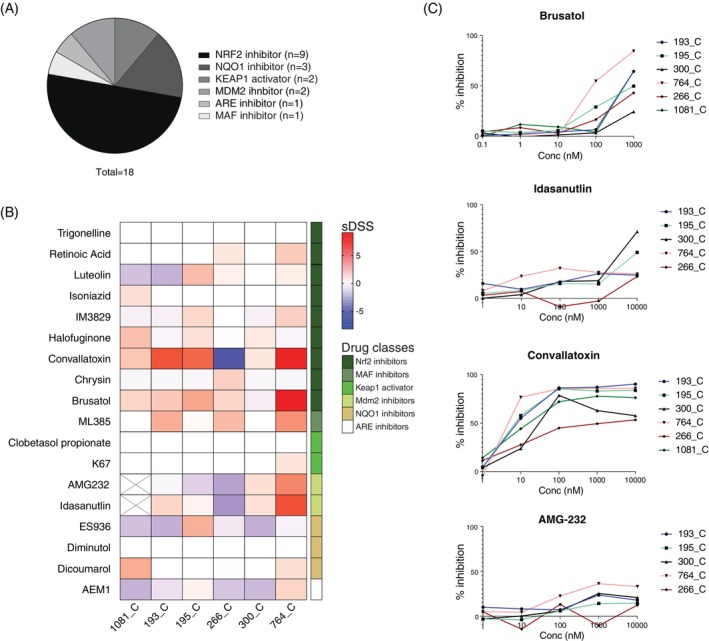
Drug screening on PDC cultures with Nrf2 inhibitors. (A) The drug library is composed by 18 compounds divided in six classes: Nrf2 inhibitors, Keap1 activator, Maf or ARE inhibitors, NQO1 and Mdm2 inhibitors. (B) Heatmap of sDSS scores in six RCC PDC cultures. Red and blue lines represent active drugs (sDSS >0) or drugs to which the PDC cultures are resistant (sDSS <0), respectively. The different drug classes are showed in green next to the heatmap. (C) Dose–response curve of the four most active compounds in pRCC cultures. The percentage of inhibition is shown in the y axis while the concentration range (5 points) is represented in the x axis.

Pharmacological inhibition of NRF2 have emerged as a promising target in cancer treatments and several small molecules have been developed to reduce NRF2 expression or activity.^29^ We performed drug screening on pRCC PDCs with 18 different NRF2‐ARE pathway inhibitors, each of them in five concentrations progressively increasing of a factor 10 (Table S5 and Figure 4A). Drug responses of six pRCC PDCs and three normal kidney PDC models were measured after 72 h incubation in the presence of the drugs by cell viability assessment (CTG). BREEZE was used to determine DSS values for each sample.^24^ The dDSS for each compound was calculated by subtracting the mean DSS of all normal cells from the DSS of each tumour PDC. Sensitivity was defined with sDSS score >5, resistance with a score <−5 (Figure 4B).

We observed that PDCs displayed distinct drug response profiles (Figure 4B). Interestingly, KEAP1‐positive activators, such as K67 or clobetasol propionate, did not show a significant effect on PDC models. In contrast, inhibition of NRF2 or its downstream target MDM2, a p53 negative regulator, was active in four samples. For example, the NRF2 inhibitor Brusatol was effective at 100 nM in 764_C and 195_C with 60% and 30% cell inhibition, respectively (Figure 4C). Interestingly, 266_C showed contrasting behaviour with different NRF2 inhibitors. While it was resistant to Convallatoxin, showing only 50% cell inhibition at a high dose (10,000 nM), the same PDC was partially sensitive to Brusatol. This difference may be attributed to the distinct mechanisms of action of these two drugs: while Convallatoxin can suppress NRF2 by modulating its proteolysis,^30^ Brusatol inhibits the NRF2 synthesis,^31^ suggesting potential differences in how these drugs interact with the NRF2 pathway or other cellular mechanisms in 266_C. In contrast, 193_C and 764_C were highly sensitive to Convallatoxin, with cytotoxicity observed at 10 nM. Interestingly, NQO1 expression was reduced in 266_C after treatment with Convallatoxin (100 nM) and ML385 (100 nM) after 24 h showing that NRF2 inhibition can bring to a downregulation of the downstream targets of NRF2‐ARE pathway (Figure S6).

Of note, 764_C, which exhibited high NQO1 activation and NQO1 protein expression, emerged as the most sensitive PDC to NRF2 pathway inhibition. This sensitivity extended beyond Convallatoxin and Brusatol, as 764_C also demonstrated a strong response to MDM2 inhibitors, such as AMG‐232 and Idasanutlin (Figure 4C). Interestingly, sample 1081 exhibited high NQO1 activity in both tumour tissue and patient‐derived cells (PDCs), with RNA‐seq data showing the highest NQO1 mRNA levels among PDCs. Real‐time PCR confirmed the overexpression of NQO1 mRNA in tumour tissue, which was further supported by elevated NQO1 protein levels compared to normal tissue. This sample demonstrated sensitivity to Dicoumarol, a competitive inhibitor of NQO1, but was resistant to ES936, an irreversible NQO1 inhibitor. Although Dicoumarol effectively inhibited NQO1 activity, Dehn et al. highlighted the limitations of this drug, including its lack of specificity and competitive inhibition mechanism,^32^ whereas ES936 is known as a potent, mechanism‐based inhibitor of NQO1.^33^ The differential response of sample 1081 to these inhibitors underscores the complexity of targeting NQO1 in cancer treatment, particularly considering the specificity and mechanism of action of the drugs.

## 4 DISCUSSION

We investigated the role of the NRF2‐ARE pathway in pRCCs and demonstrated the activation of NRF2‐ARE pathway, based on IHC, mRNA level and enzymatic assay of NQO1 in six MET‐independent pRCC patients. WES analysis of 60 pRCC specimens revealed six tumours (10%) with mutations in the four key genes, *NFE2L2*, *KEAP1*, *CUL3* and *BACH1*, of the NRF2‐ARE pathway. mRNA and protein expression analysis of NQO1 confirmed the activation of the NRF2‐ARE pathway in pRCC tumour tissues and in the paired PDCs. Drug screening with several NRF2‐ARE pathway inhibitors showed that Brusatol and Convallatoxin, targeting Nrf2, are active on pRCC PDCs with aberrant NRF2‐ARE pathway activity.

The consequence of an aberrant regulation of the NRF2‐ARE pathway is the constitutive NRF2 activation leading to cellular resiliency to various stressors.^34^, ^35^ Using STRING database, we identified 15 genes encoding for the main upstream complex of the NRF2‐ARE pathway such as NRF2, Keap1 and Cul3 and other interactor partners that are involved in NRF2 nuclear translocation and transcription of target genes. Next, we analysed the genetic alterations of these genes by WES. We identified one *NFE2L2* missense variant (c.246A > C) mutation described as pathogenic in the COSMIC database (COSV67960116).^36^ This mutation lies in *NFE2L2* exon 2, a region encoding for the Neh2 domain, which contains two highly conserved motifs (DLG and ETGE) responsible for KEAP1 binding.^37^, ^38^ Mutations in *NFE2L2* exon 2 were already described as a common mechanism of NRF2 hyper‐activation in cancer because alterations in this exon lead to an aberrant Neh2 domain and consequently to NRF2 stabilization in the cytoplasm by escaping KEAP1‐regulated degradation.^39^

Mutations in *CUL3* and *BACH1* were also identified in four samples. Cul3 is a the scaffold component of the E3 ligase‐complex and regulates NRF2 degradation by ubiquitination.^40^, ^41^ Missense mutations can interfere with the ubiquitin activity of Cul3 leading to abnormal NRF2 accumulation. Interestingly, somatic mutations in *NFE2L2* and *CUL3* accompanied by NRF2 activation were previously described in sporadic pRCC confirming that NRF2 is positively activated in a subset of pRCCs.^14^ While we found 5% of our sample harbouring MET mutations, the NRF2‐ARE pathway mutated samples did not show *MET* mutations, suggesting that the NRF2‐ARE pathway can represent the main *MET*‐independent pathway in pRCC. Mutation frequencies in the four NRF2‐ARE pathway genes observed in our pRCC cohort (12%) and in the TCGA dataset (8%), are suggesting that the NRF2‐ARE pathway is mutationally affected in approximately 10% of pRCC. Interestingly, NQO1 upregulations was already described in many cancers such as pancreatic cancer, uterine cervical cancer, melanoma, and lung cancer.^6^, ^42^ High‐level expression of NQO1 protein was correlated with late clinical stage in breast cancer.^43^ Our analysis of NQO1 expression in 638 RCCs showed NQO1 positivity in one‐third of pRCC while only minor fractions of ccRCCs and chRCCs showed cytoplasmic NQO1 staining. This result provides further evidence that NRF2‐ARE pathway activation is especially relevant for pRCC. Importantly, NQO1 expression was an independent prognostic parameter in multivariate analysis including pRCC and ccRCC subtypes.

To study the functional deregulation of the NRF2‐ARE pathway in patient‐derived in vitro models by NQO1 mRNA and protein expression, we generated PDCs from nine pRCC patients.^19^ Five of nine PDCs demonstrated high activity as well as high mRNA and protein levels of NQO1 that were comparable to the paired tumours. These findings confirmed our TMA data, which showed a preferential NRF2 hyper‐activity in high grade, and late stage pRCCs correlating with worse patient outcome. Differential gene expression analysis from RNA‐seq further confirmed these observations, revealing that NQO1 had the highest log2 fold change among other key NRF2‐ARE pathway genes. Notably, three PDCs showed high transcripts per million (TPM) for NQO1, and two PDCs for MDM2. Interestingly, 1081_C and 193_C, which had the highest TPM for NQO1, also harboured missense mutations in KEAP1 and NFE2L2, suggesting a potential link between these genomic alterations and NQO1 overexpression.

Currently, there is limited efficacy of targeted treatments for metastatic pRCC. Phase II/III data demonstrated activity of MET‐TKI Savolitinib in MET‐driven pRCC.^44^ However, impact of MET status on treatment outcomes in pRCC is controversial. In previous clinical trials, MET upregulation was defined as *MET* and/or *HGF* amplification, chromosome 7 copy number gain (the gene locus of both *MET* and *HGF*), and/or *MET* kinase domain mutations. Using this definition, MET upregulation is reported in up to 80% of pRCC.^7^ Importantly, chromosome 7 copy number gains are regarded as frequent and unspecific in RCC tumorigenesis^45^ and *MET* gene mutations are rather rare in pRCC (<10%; www.cbioportal.org). *MET* amplifications are found only in 1.8% of pRCC TCGA dataset. In this study, we identified *MET* mutations in 5% and chromosome 7 copy number gains in 74.3% in our cohort of pRCC. Choueiri et al. have reported in a phase II study of advanced pRCC that about 40% of pRCC are *MET*‐driven and 40% *MET*‐independent.^8^ Albigels et al. have also described high rates of chromosome 7 gains (42%), but only 7% *MET* mutations and 6% *MET* amplifications.^46^ Therefore, relevance of MET status as predictive biomarker in MET‐driven pRCC needs to be clarified in future clinical trials.

NRF2‐ARE pathway may be activated in a significant number of *MET*‐independent pRCC, because 33% of pRCC were NQO1 positive in our TMA cohort. NQO1 activation in our pRCC tissue samples and PDCs let us speculate that targeting the NRF2‐ARE pathway could be a potential therapeutical option in pRCC. Based on literature data, we selected 18 compounds targeting the NRF2‐ARE pathway. Comprehensive drug screening using PDCs model from six patients revealed their sensitivity to two NRF2 inhibitors, Brusatol and Convallatoxin, with partial responses in five PDCs. Brusatol was shown to reduce NRF2 protein levels in PDX adenocarcinoma mouse models.^47^, ^48^ Moreover, Brusatol‐mediated inhibition of NRF2 sensitized non‐small cell lung cancer cells to cisplatin treatment.^49^ Drug sensitivity to Convallatoxin was observed in three PDCs. PDCs from one patient (193_C) with high NQO1 mRNA level and protein expression showed highest response to these inhibitors. In contrast, PDCs (266_C) with low NQO1 RNA level and almost basal activity of NQO1 were resistant to the drug. Interestingly, this PDC showed also resistance to NQO1 and MDM2 inhibitors. Recent studies have highlighted the distinct cytotoxic effects of MDM2 and NQO1 inhibitors in various cancer models. MDM2 inhibitors primarily induce cell death by disrupting the MDM2‐p53 interaction, leading to the activation of p53‐dependent apoptotic pathways and cell cycle arrest.^50^ On the other hand, NQO1 inhibitors, like Dicoumarol, induce cytotoxicity by disrupting cellular redox homeostasis and increasing oxidative stress, which is particularly detrimental in tumour cells overexpressing NQO1.^51^ Our results highlight NQO1 and MDM2 inhibition as a promising therapeutic approach for high NQO1‐expressing pRCC. Notably, two PDCs with NQO1 basal activity were resistant to all tested compounds supporting the link between the NRF2 pathway activation status and its druggability.

Only a minor part of the NRF2‐ARE pathway inhibitors showed the expected effects in our PDCs. The conditions and the drug concentration range chosen in our experiments may not be optimal for some of these drugs. For example, Diminutol, a compound able to reduce NQO1 activity in pancreatic cancer cell lines,^52^ did not show any effects on our PDCs suggesting that further studies are needed to test different concentrations and possible responses.

Targeting the NRF2‐ARE pathway could also represent a promising strategy to target a subgroup of ccRCC patients showing aberrations in this pathway. Notably, previous study showed that NQO1 inhibits the proteasomal degradation of HIF‐1α,^53^ a key driver in ccRCC tumorigenesis.^54^ We also identified that high‐level expression of NQO1 in ccRCC is associated with poor prognosis. Additionally, mutations in *CUL3* or *NFE2L2* are present in about 1% of ccRCC tumours according to TCGA data. These results suggest that inhibition of the NRF2‐ARE pathway could represent a viable therapeutic strategy for this subset of ccRCC patients.

In conclusion, pRCC is characterised by aberration in the NRF2‐ARE pathway in a subset of aggressive tumours. Our results further suggest NRF2‐ARE pathway inhibitors as a promising new alternative for the treatment of patients with advanced pRCC.

## AUTHOR CONTRIBUTIONS

**Silvia Angori:** Conceptualization; funding acquisition; methodology; validation; writing – original draft; writing – review and editing. **Harini Lakshminarayanan:** Investigation; methodology; writing – review and editing. **Amir Banaei‐Esfahani:** Formal analysis; methodology; software; visualization. **Katharina Mühlbauer:** Data curation; investigation; methodology. **Hella Anna Bolck:** Data curation; methodology. **Olli Kallioniemi:** Investigation; resources. **Vilja Pietiäinen:** Investigation; resources; writing – original draft; writing – review and editing. **Peter Schraml:** Conceptualization; funding acquisition; methodology; project administration; resources; supervision; writing – original draft; writing – review and editing. **Holger Moch:** Conceptualization; funding acquisition; project administration; resources; supervision; writing – original draft; writing – review and editing.

## FUNDING INFORMATION

This study was supported by the University Research Priority Program (URPP) in Translational Cancer Research (University of Zurich, Switzerland), the SAKK_Amgen Research Grant funded by Amgen to support projects for outstanding and novel translational cancer research and the Swiss National Science Foundation grant (No. S‐87701‐03‐01).

## CONFLICT OF INTEREST STATEMENT

The authors declare no potential conflicts of interest.

## ETHICS STATEMENT

The Tissue Biobank at the University Hospital of Zurich (Switzerland) provided the tissue samples. All patients provided written consent for this study, which was authorized by the local ethics committee in Canton Zurich (BASEC 2019–01959).

## Supporting information


**DATA S1.** Supporting information.

**TABLE S6.** WES sequencing coverage and quality statistics.

**TABLE S7.** RNA‐seq sequencing coverage and quality statistics.

## Data Availability

The WES data that support the study are available in cBioPortal at https://pubmed.ncbi.nlm.nih.gov/22588877/ (DOI: 10.1158/2159‐8290.CD‐12‐0095) reference dataset: Kidney Renal Papillary Cell Carcinoma (TCGA, PanCancer Atlas). Sequencing data from patient samples were submitted on the SRA (NCBI) depository (Project ID: PRJNA932209). Other data that support the findings of this study are available from the corresponding author upon request.
