# Supplementary material for: Exploiting NRF2‐ARE pathway activation in papillary renal cell carcinoma
Source: Int J Cancer. 2024 Dec 20;156(7):1457–69. doi: 10.1002/ijc.35311 (PMC11789458; doi:10.1002/ijc.35311)
Supplement: Supplementary file 1 — DATA S1. Supporting information. [file IJC-156-1457-s003.pdf]

Supplementary information

Exploiting NRF2-ARE Pathway Activation in Papillary Renal Cell Carcinoma

Silvia Angori, Harini Lakshminarayanan, Amir Banaei-Esfahani, Katharina Mühlbauer, Hella Anna Bolck, Olli Kallioniemi, Vilja Pietiäinen, Peter Schraml, Holger Moch

Table of content:

- 5 supplementary tables
- 6 supplementary figures
- 1 supplementary table: WES sequencing coverage and quality statistics (available in a separate file named supplementary table 6)
- 1 supplementary table: RNA-seq sequencing coverage and quality statistics (available in a separate file named supplementary table 7)

Supplementary Tables

Supplementary Table 1. NQO1 expression in ccRCC correlates with high ISUP grade but not with tumour stage.

	NQO1 negative N (%)	NQO1 positive* N (%)	P value
Grade 1	14 (100)	0 (0)	0.0045
Grade 2	159 (95.8)	7 (4.2)	
Grade 3	138 (93.9)	9 (6.1)	
Grade 4	120 (85.7)	20 (14.3)	
pT 1-2	245 (92.8)	19 (7.2)	n.s.
pT 3-4	180 (91.4)	17 (8.6)	

*Tumour stage was not available from 4 of 40 NQO1 positive ccRCC.

Supplementary Table 2: Multivariate Cox regression analysis in ccRCC and pRCC including NQO1 expression and prognostic pathological parameters tumor grade and stage.

Variable	Characteristics	Recurrence-free survival		
		HR	95% CI	P-value
Tumor stage	pT1/2 vs pT3/4	2.604	1.899-3.572	<.001
ISUP grade	1, 2, 3, 4	1.774	1.249-2.521	.001
NQO1	Negative vs positive	1.726	1.162-2.565	.007

Supplementary Table 3: Mutations in the key genes of the NRF2-ARE pathway identified in a subset of pRCC tumours. (Chr: chromosome, Gene: gene name, Cosmic_ID: code assigned by COSMID identifying a unique mutation in the database, Ref: reference, letter based on the reference genome, Mut: mutation characterising our sample, Mut. Type: type of mutation; Sample ID: unique code assigned to each sample in our analysis)

Chr	Gene	Cosmic_ID	Ref	Mut	Mut. Type	Sample
chr21	*BACH1*	.	A	T	missense_variant	474_T
chr2	*CUL3*	.	C	A	stop_gained	28_T
chr2	*CUL3*	.	C	A	missense_variant	6_T
chr19	*KEAP1*	.	A	C	missense_variant	5_T
chr19	*KEAP1*	.	A	G	missense_variant	1081_C
chr19	*KEAP1*	.	A	G	missense_variant	1081_T
chr2	*NFE2L2*	COSV67960116	T	G	missense_variant	193_C
chr2	*NFE2L2*	COSV67960116	T	G	missense_variant	193_T

Supplementary Table 4: List of NRF2-regulated genes analysed by RNA-seq with log2 foldchange and p value.

Gene	log2FoldChange	pvalue
NQO1	2,63840296	9,419E-06
HMOX2	0,75623486	0,01133057
PRKCA	1,20651954	0,02809338
JUN	-0,6663808	0,04767806
RBX1	1,11209393	0,05519556
GSTA2	-1,855059	0,08059885
NEDD8	0,78009876	0,08550257
CUL3	-0,267705	0,15699501
MAFK	0,79725193	0,26070934
NFE2L2	-0,2847042	0,43510618
HMOX1	-0,4774124	0,47936844
BACH1	0,34498981	0,52838814
CRYZ	-0,1942764	0,66109634
MAFG	0,14929042	0,67109322
KEAP1	0,07774991	0,71306399
MDM2	-0,0731743	0,82869126
MAFF	-0,0558205	0,91551978

Supplementary Table 5: List of compounds used in the drug screening. These compounds can be subdivided in NRF2 inhibitors, NRF2 target bene inhibitors, Keap1 positive regulators, NQO1 and Mdm2 inhibitors. In the table, effects and mechanism of actions for each compound are shown. In the last column, the experimental model where these drugs have been tested previously.

Class	Effects	Mechanism	Model
NRF2 Inhibitors			
1. **Flavonoids**			
Luteolin	NRF2 inhibitor	Reduction of mRNA and protein levels of NRF2	mice transplanted with human lung carcinoma epithelial cells [1], [2]
Chrysin	NRF2 inhibitor	Down-regulation through PI3K/AKT pathway	human hepatocellular carcinoma cells [3]
2. **Antitubercular Drugs**			
Isoniazid	NRF2 inhibitor	Inhibition of NRF2-inducible genes	human hepatocellular carcinoma cells [4]
3. **Vitamins**			
Retinoic acid	NRF2 inhibitor	Binding of NRF2 in ARE sequence binding domain	acute myeloid leukemia cells [5], [6]
4. **Others**			
Brusatol	NRF2 inhibitor	Reduction of NRF2 protein level	mice transplanted with adenocarcinomic alveolar epithelial cells [2], [5], [8]
Trigonelline	NRF2 inhibitor	Reduction of NRF2 nuclear import	pancreatic adenocarcinoma cells [9], [10]
Convallatoxin	NRF2 inhibitor	Suppression of NRF2	lung cancer cells [12]
Halofuginone	NRF2 inhibitor	Suppression of NRF2 synthesis	liver hepatocellular carcinoma [13], [14]
IM3829	NRF2 inhibitor	Block of NRF2 nuclear translocation	human lung cancer cell lines [15]
NRF2 target gene inhibitors			
AEM1	NRF2-ARE target genes inhibition	Reduction of mRNA and protein levels of NRF2-ARE target genes	mice transplanted with adenocarcinomic alveolar epithelial cells [7]
ML385	NRF2-MAF binding inhibitor	Inhibition of the NRF2-MAF binding	lung cancer cells [2], [11]
Keap1 Positive Regulators			
K67	Keap1 activator	Reduction of the interaction between p62 and Keap1	hepatocellular carcinoma cells [2], [16], [17]
Clobetasol Propionate	Glucocorticoid	Induction of NRF2 degradation	lung cancer cells [18], [19]
NQO1 Inhibitors			
Diminutol	NQO1 inhibitor	Inhibition of NQO1 activity	human pancreatic cancer cells [20]
Dicoumarol	NQO1 inhibitor	Inhibition of NQO1 activity	solid tumours [21], [22]
ES936	NQO1 inhibitor	Bind of NQO1 irreversibly	human pancreatic cancer cells [23]
Mdm2 Inhibitors			
Idasanutlin	Mdm2/p53 interaction inhibitor	Inhibition of p53–Mdm2 interaction	acute myeloid leukemia cells [24], [25]
AMG232	Mdm2/p53 interaction inhibitor	p53 activity restored	clinical trial for glioblastoma [25], [26]

Supplementary Figures

Supplementary Figure 1: NQO1 expression in pRCC and ccRCC correlates with patient survival. NQO1 (1) = expression and NQO1 (0) = no expression.

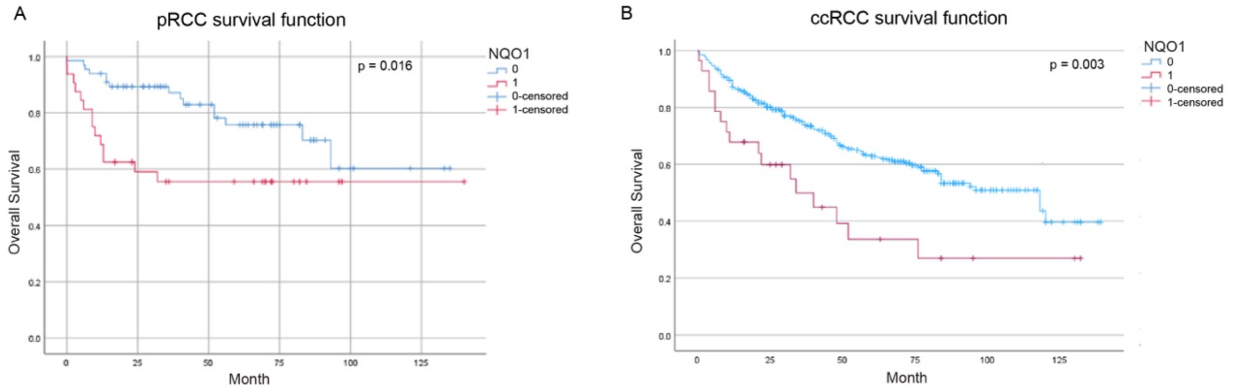

Supplementary Figure 2: Copy number variation profiles of 60 pRCC samples. Red and blue squares indicate the region characterised by chromosomal gain or loss, respectively, according to the Nexus Express Software with p-value < 0.05 and aggregate % cut-off = 35.0. Gain of chromosomes 7, 16 and 17 are the most frequent alteration in pRCC. Our cohort showed also less frequent gain of chromosomes 2, 3, 12 and 20.

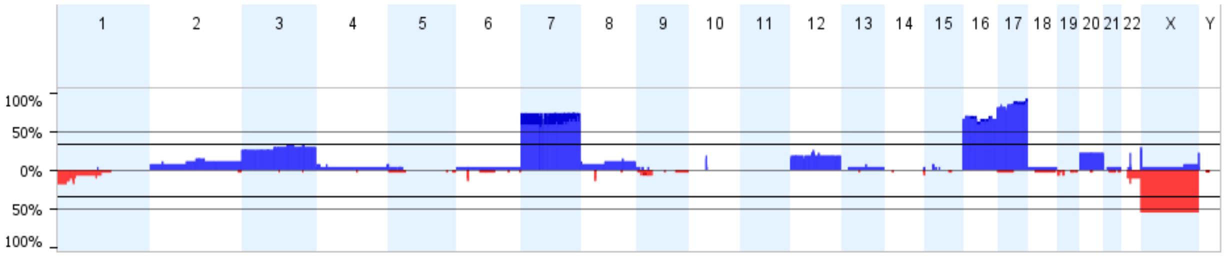

Supplementary Figure 3: NRF2 expression in pRCCs. (A) qPCR analysis of *NFE2L2* in pRCCs (n =9). The relative expression was normalised based on the matched normal kidney tissue (when present) or on the average values of normal tissue. The plot shows the average value of three independent replicates (*** = 3 replicates) (B) Immunohistochemistry analysis of NRF2 in pRCC tumour and paired normal tissues. The images were taken with 10X objective (the same images for samples 193 and 266 have been shown in Figure 3).

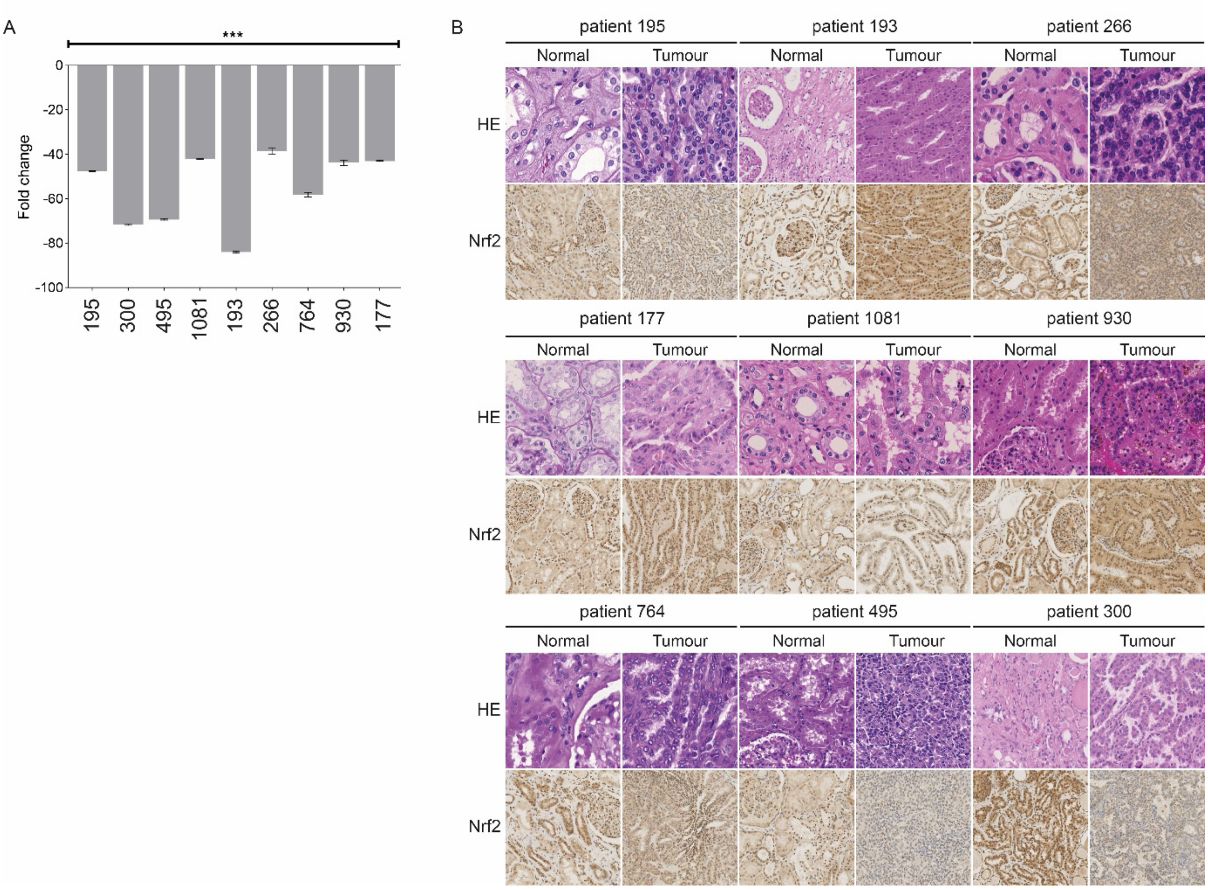

Supplementary Figure 4: Immunohistochemistry analysis of NQO1. NQO1 over-expression in the five tumour tissues and PDCs 474, 193, 764, 177 and 1081. For each patient sample, fixed sections from FFPE blocks of normal, tumour and patient-derived cell culture (when available) were stained for NQO1 and for haematoxylin and eosin. The images were taken with 10X objective (the same images for samples 193 and 266 have been shown in Figure 3).

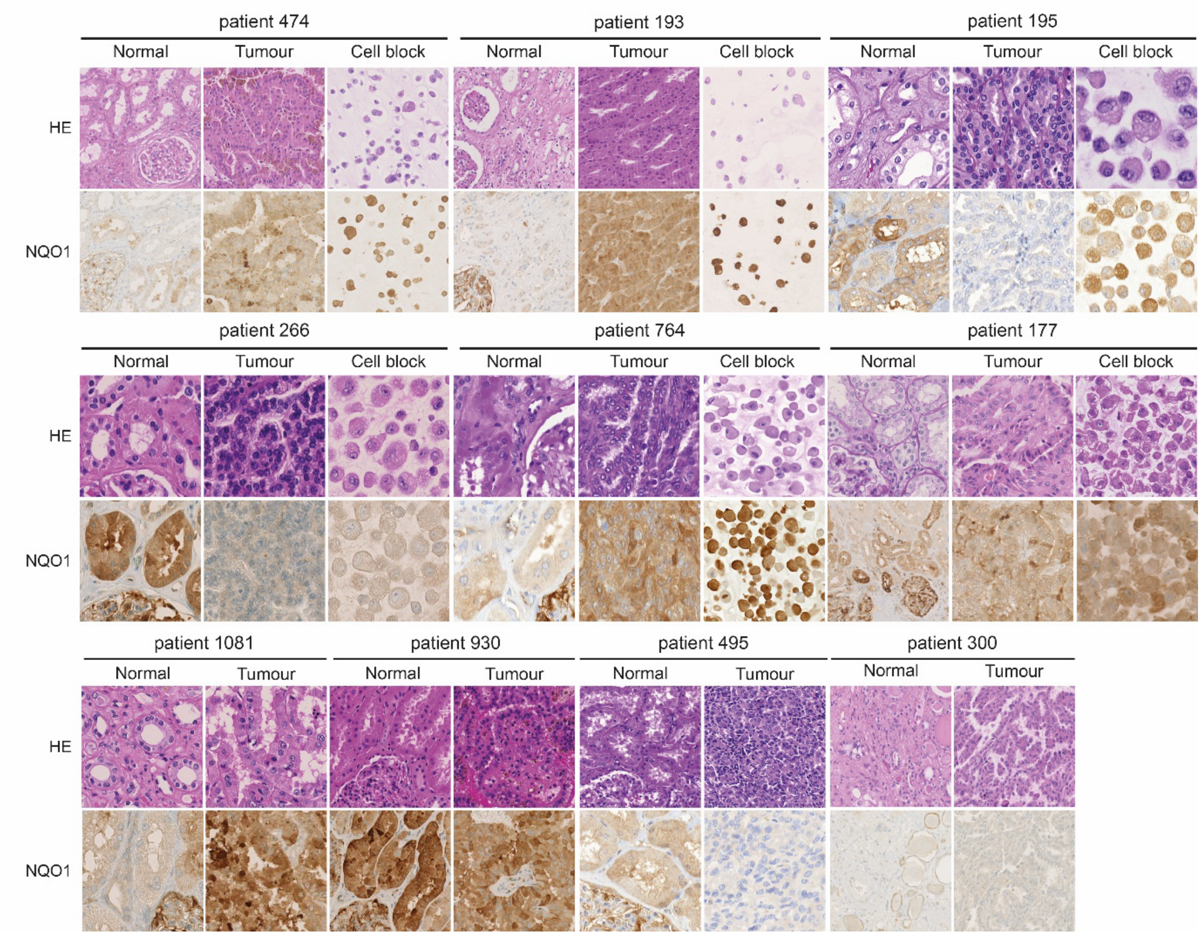

Supplementary Figure 5: Transcript per Million (TPM) expression levels of key genes in the NRF2-ARE pathway in tumor PDCs versus normal PDCs. The plots display TPM values for genes NFE2L2, KEAP1, CUL3, BACH1, NQO1 and MDM2. Tumour and normal PDCs are showed in red and grey, respectively. Each dot represents one PDC sample.

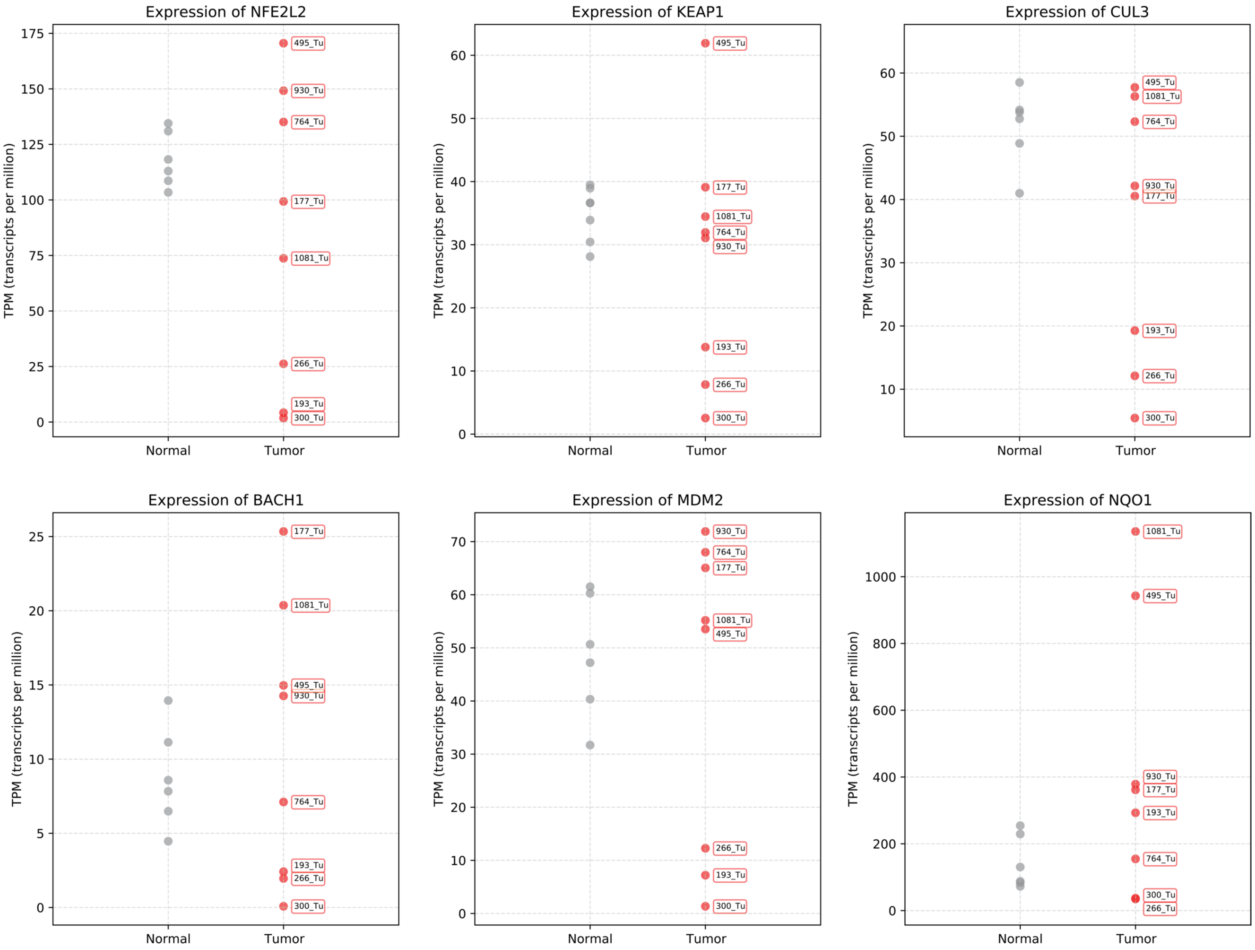

Supplementary Figure 6: Drug treatment on PDCs. Three PDCs (193_C, 266_C and 195_C) were treated with three NRF2 inhibitors: Brusatol, Convallatoxin and ML385 for 24 hours. The expression levels of MDM2 (panel A) and NQO1 (panel B) were normalized to tubulin and compared to the DMSO control condition. Cell viability (panel C) was normalized to the initial number of seeded cells (500,000 cells).

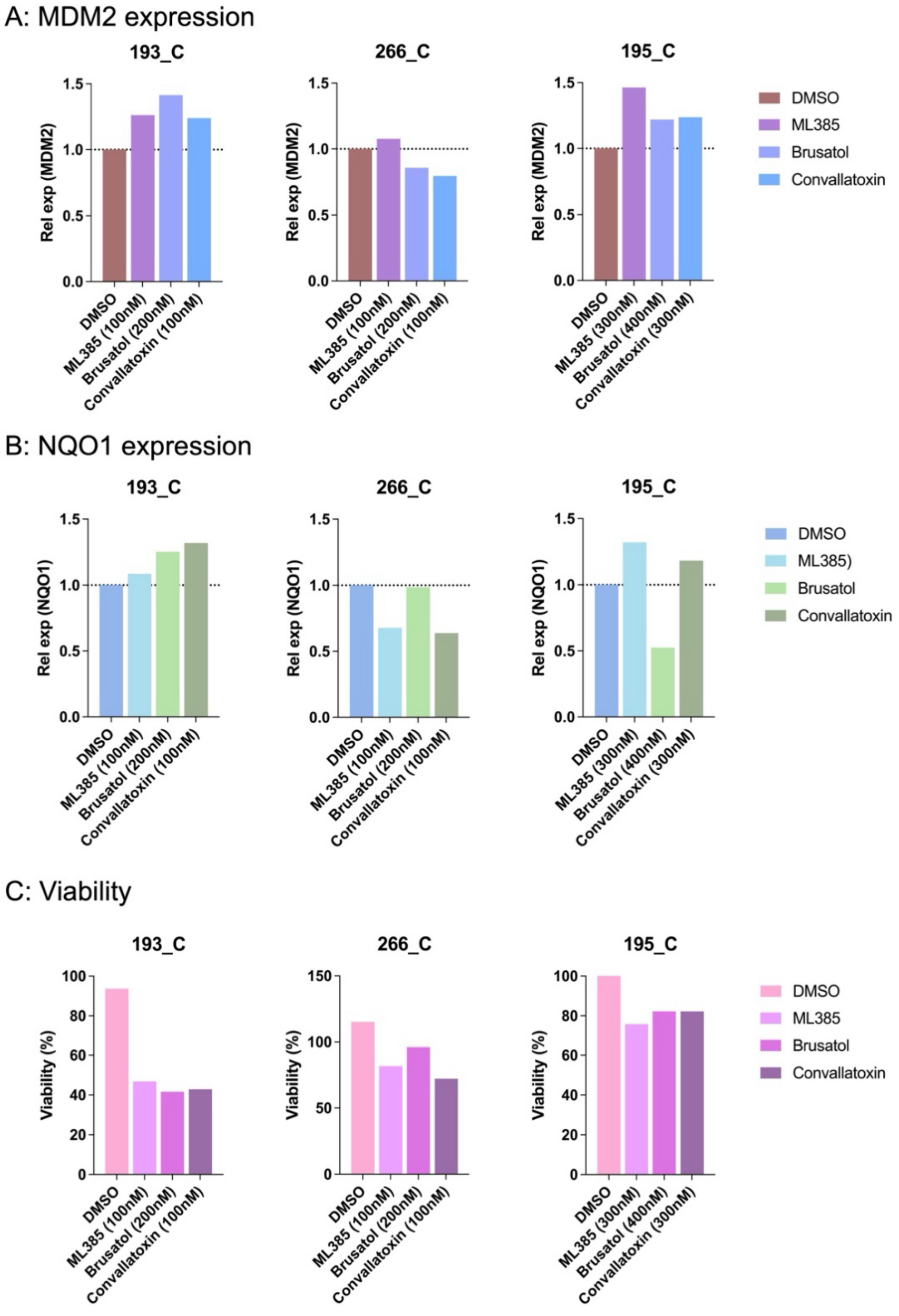

Additional references

[1] X. Tang *et al.*, 'Luteolin inhibits NRF2 leading to negative regulation of the NRF2/ARE pathway and sensitization of human lung carcinoma A549 cells to therapeutic drugs', *Free Radical Biology and Medicine*, vol. 50, no. 11, pp. 1599–1609, Jun. 2011, doi: 10.1016/j.freeradbiomed.2011.03.008.

[2] E. Panieri and L. Saso, 'Potential Applications of NRF2 Inhibitors in Cancer Therapy', *Oxidative Medicine and Cellular Longevity*, vol. 2019, p. e8592348, Apr. 2019, doi: 10.1155/2019/8592348.

[3] A.-M. Gao, Z.-P. Ke, F. Shi, G.-C. Sun, and H. Chen, 'Chrysin enhances sensitivity of BEL-7402/ADM cells to doxorubicin by suppressing PI3K/Akt/NRF2 and ERK/NRF2 pathway', *Chemico-Biological Interactions*, vol. 206, no. 1, pp. 100–108, Oct. 2013, doi: 10.1016/j.cbi.2013.08.008.

[4] A. K. Verma *et al.*, 'Isoniazid prevents NRF2 translocation by inhibiting ERK1 phosphorylation and induces oxidative stress and apoptosis', *Redox Biology*, vol. 6, pp. 80–92, Dec. 2015, doi: 10.1016/j.redox.2015.06.020.

[5] J. Zhu *et al.*, 'An overview of chemical inhibitors of the NRF2-ARE signaling pathway and their potential applications in cancer therapy', *Free Radical Biology and Medicine*, vol. 99, pp. 544–556, Oct. 2016, doi: 10.1016/j.freeradbiomed.2016.09.010.

[6] X. J. Wang, J. D. Hayes, C. J. Henderson, and C. R. Wolf, 'Identification of retinoic acid as an inhibitor of transcription factor NRF2 through activation of retinoic acid receptor alpha', *PNAS*, vol. 104, no. 49, pp. 19589–19594, Dec. 2007, doi: 10.1073/pnas.0709483104.

[7] M. J. Bollong, H. Yun, L. Sherwood, A. K. Woods, L. L. Lairson, and P. G. Schultz, 'A Small Molecule Inhibits Deregulated NRF2 Transcriptional Activity in Cancer', *ACS Chem. Biol.*, vol. 10, no. 10, pp. 2193–2198, Oct. 2015, doi: 10.1021/acschembio.5b00448.

[8] J. H. Lee *et al.*, 'Brusatol, a NRF2 Inhibitor Targets STAT3 Signaling Cascade in Head and Neck Squamous Cell Carcinoma', *Biomolecules*, vol. 9, no. 10, Sep. 2019, doi: 10.3390/biom9100550.

[9] A. E. Ghule, S. S. Jadhav, and S. L. Bodhankar, 'Trigonelline ameliorates diabetic hypertensive nephropathy by suppression of oxidative stress in kidney and reduction in renal cell apoptosis and fibrosis in streptozotocin induced neonatal diabetic (nSTZ) rats', *Int. Immunopharmacol.*, vol. 14, no. 4, pp. 740–748, Dec. 2012, doi: 10.1016/j.intimp.2012.10.004.

[10] A. Arlt *et al.*, 'Inhibition of the NRF2 transcription factor by the alkaloid trigonelline renders pancreatic cancer cells more susceptible to apoptosis through decreased proteasomal gene expression and proteasome activity', *Oncogene*, vol. 32, no. 40, Art. no. 40, Oct. 2013, doi: 10.1038/onc.2012.493.

[11] A. Singh *et al.*, 'Small molecule inhibitor of NRF2 selectively intervenes therapeutic resistance in KEAP1-deficient NSCLC tumors', *ACS Chem Biol*, vol. 11, no. 11, pp. 3214–3225, Nov. 2016, doi: 10.1021/acschembio.6b00651.

[12] J. Lee, J.-S. Kang, L. B. Nam, O.-K. Yoo, and Y.-S. Keum, 'Suppression of NRF2/ARE by convallatoxin sensitises A549 cells to 5-FU-mediated apoptosis', *Free Radic. Res.*, vol. 52, no. 11–12, pp. 1416–1423, Dec. 2018, doi: 10.1080/10715762.2018.1489132.

[13] K. Tsuchida *et al.*, 'Halofuginone enhances the chemo-sensitivity of cancer cells by suppressing NRF2 accumulation', *Free Radic. Biol. Med.*, vol. 103, pp. 236–247, 2017, doi: 10.1016/j.freeradbiomed.2016.12.041.

[14] H. Lin *et al.*, 'Small molecular NRF2 inhibitors as chemosensitizers for cancer therapy', *Future Med Chem*, vol. 12, no. 3, pp. 243–267, 2020, doi: 10.4155/fmc-2019-0285.

[15] S. Lee *et al.*, 'An effective strategy for increasing the radiosensitivity of Human lung Cancer cells by blocking NRF2-dependent antioxidant responses', *Free Radical Biology and Medicine*, vol. 53, no. 4, pp. 807–816, Aug. 2012, doi: 10.1016/j.freeradbiomed.2012.05.038.

[16] D. Yasuda *et al.*, 'Inhibitors of the protein–protein interaction between phosphorylated p62 and Keap1 attenuate chemoresistance in a human hepatocellular carcinoma cell line', *Free Radical Research*, vol. 0, no. 0, pp. 1–13, Feb. 2020, doi: 10.1080/10715762.2020.1732955.

[17] T. Saito *et al.*, 'p62/Sqstm1 promotes malignancy of HCV-positive hepatocellular carcinoma through NRF2-dependent metabolic reprogramming', *Nature Communications*, vol. 7, no. 1, Art. no. 1, Jun. 2016, doi: 10.1038/ncomms12030.

[18] B.-J. Jung, H.-S. Yoo, S. Shin, Y.-J. Park, and S.-M. Jeon, 'Dysregulation of NRF2 in Cancer: from Molecular Mechanisms to Therapeutic Opportunities', *Biomol Ther (Seoul)*, vol. 26, no. 1, pp. 57–68, Jan. 2018, doi: 10.4062/biomolther.2017.195.

[19] E.-J. Choi *et al.*, 'A clinical drug library screen identifies clobetasol propionate as an NRF2 inhibitor with potential therapeutic efficacy in KEAP1 mutant lung cancer', *Oncogene*, vol. 36, no. 37, pp. 5285–5295, 14 2017, doi: 10.1038/onc.2017.153.

[20] S. M. Wignall *et al.*, 'Identification of a Novel Protein Regulating Microtubule Stability through a Chemical Approach', *Chemistry & Biology*, vol. 11, no. 1, pp. 135–146, Jan. 2004, doi: 10.1016/j.chembiol.2003.12.019.

[21] B. Buranrat, A. Prawan, U. Kukongviriyapan, S. Kongpetch, and V. Kukongviriyapan, ‘Dicoumarol enhances gemcitabine-induced cytotoxicity in high NQO1-expressing cholangiocarcinoma cells’, *World J Gastroenterol*, vol. 16, no. 19, pp. 2362–2370, May 2010, doi: 10.3748/wjg.v16.i19.2362.
[22] M. Stiborova *et al.*, ‘Dicoumarol inhibits rat NAD(P)H:quinone oxidoreductase in vitro and induces its expression in vivo’, *Neuro Endocrinol. Lett.*, vol. 35 Suppl 2, pp. 123–132, 2014.
[23] D. L. Dehn, D. Siegel, E. Swann, C. J. Moody, and D. Ross, ‘Biochemical, Cytotoxic, and Genotoxic Effects of ES936, a Mechanism-Based Inhibitor of NAD(P)H:quinone Oxidoreductase 1, in Cellular Systems’, *Mol Pharmacol*, vol. 64, no. 3, pp. 714–720, Sep. 2003, doi: 10.1124/mol.64.3.714.
[24] C. Lehmann, T. Friess, F. Birzele, A. Kiialainen, and M. Dangl, ‘Superior anti-tumor activity of the MDM2 antagonist idasanutlin and the Bcl-2 inhibitor venetoclax in p53 wild-type acute myeloid leukemia models’, *J Hematol Oncol*, vol. 9, Jun. 2016, doi: 10.1186/s13045-016-0280-3.
[25] M. Konopleva *et al.*, ‘MDM2 inhibition: an important step forward in cancer therapy’, *Leukemia*, pp. 1–17, Jul. 2020, doi: 10.1038/s41375-020-0949-z.
[26] J. Canon *et al.*, ‘The MDM2 Inhibitor AMG 232 Demonstrates Robust Antitumor Efficacy and Potentiates the Activity of p53-Inducing Cytotoxic Agents’, *Mol Cancer Ther*, vol. 14, no. 3, pp. 649–658, Mar. 2015, doi: 10.1158/1535-7163.MCT-14-0710.
